# A case of Aromatase deficiency due to a novel *CYP19A1* mutation

**DOI:** 10.1186/1472-6823-14-16

**Published:** 2014-02-19

**Authors:** Lucia Gagliardi, Hamish S Scott, Jinghua Feng, David J Torpy

**Affiliations:** 1 Endocrine and Metabolic Unit, Royal Adelaide Hospital, North Terrace, Adelaide, SA 5000, Australia; 2 Department of Genetics and Molecular Pathology, Centre for Cancer Biology, SA Pathology, Frome Road, Adelaide, SA 5000, Australia; 3 School of Medicine, University of Adelaide, Adelaide, SA 5000, Australia; 4 Cancer Genomics Facility, Centre for Cancer Biology, SA Pathology, Adelaide, SA 5000, Australia; 5 School of Molecular and Biomedical Science, University of Adelaide, Adelaide, SA 5000, Australia; 6 Division of Health Sciences, School of Pharmacy and Medical Sciences, University of South Australia, Adelaide, SA 5000, Australia

**Keywords:** Aromatase deficiency, Pubertal development, Streak ovaries, Androgens

## Abstract

**Background:**

Aromatase deficiency is a rare, autosomal recessive disorder of which there are approximately twenty four case reports. The aromatase enzyme is crucial in the biosynthesis of oestrogens from androgens. The phenotype of aromatase deficiency therefore is the result of androgen excess and oestrogen deficiency in the absence of normal aromatase activity. We report the first case of aromatase deficiency diagnosed in a female adult, at the age of 32 years, due to a novel duplication in the aromatase gene.

**Case presentation:**

A 32 year old Indian woman presented with a history of gender assignment difficulties at birth, lack of pubertal development, osteopaenia with fracture and tall stature. She had central obesity, impaired fasting glucose and borderline hypertension. Past examinations had revealed partial fusion of urethra and vagina, hypoplastic uterus and streak ovaries. The ovaries had been excised due to malignant risk after an initial clinical diagnosis of Turner’s syndrome with Y mosaicism. Oestrogen replacement commenced shortly after her fracture, in adulthood. After reassessment, aromatase deficiency was diagnosed. Sequencing of the coding exons of the aromatase (*CYP19A1*; OMIM 109710) gene revealed a novel 27-base duplication in exon 8 (p.Ala306_Ser314*dup*). This duplication, occurring within the aromatase α-helix, would be likely to disrupt substrate (androgen) and cofactor (protoporphyrin IX) binding, resulting in a lack of oestrogen synthesis.

**Conclusions:**

We report a female with a phenotype compatible with aromatase deficiency which was unrecognised until adulthood and found she had a novel duplication in *CYP19A1*. Previous case reports have described polycystic ovarian morphology, especially in childhood and adolescence, but never streak ovaries. This may reflect the few adult cases reported, that aromatase deficiency in females is generally diagnosed at birth and oestrogen treatment commences decades earlier than occurred in our patient. Streak ovaries are consistent with the phenotype of the aromatase knockout mouse followed through adulthood. The observed clinical features of obesity, dysglycaemia and hypertension, are compatible with the observation that lack of a counterbalancing effect of oestrogen on tissue androgens until adulthood may lead to a metabolic syndrome phenotype. This report broadens the spectra of phenotype and genetic mutations underlying this rare disorder.

## Background

Human aromatase is a 58KDa protein which was first isolated from the placenta in the 1980s [1]. Tissues expressing aromatase include the granulosa cells and lutea corpora of the ovary, Leydig and Sertoli cells of the testis, breast, syncytiotrophoblast of the placenta, neurons, brain (including hypothalamus), liver, pre-adipocytes and fibroblasts, vascular smooth muscle cells, chondrocytes and osteoblasts [2]. Aromatase is a cytochrome P450 critical in the biosynthesis of oestrogen since it converts androgenic substrates, primarily testosterone and androstenedione, to oestradiol and oestrone, respectively [2]. In pregnancy, placental aromatisation of 16-hydroxy-dehydroepiandrosterone sulphate, arising from foetal liver hydroxylation of dehydroepiandrosterone sulphate produced by the foetal adrenal, is the major source of circulating oestrogens; the activity of placental aromatase protects the foetus against the virilising action of foetal androgens [3]. Placental aromatisation of foetal androgens also prevents high levels from reaching the maternal circulation, where they would result in maternal virilisation during pregnancy, subsiding post-partum.

The single gene encoding human aromatase (*CYP19A1;* OMIM 109710) is a 120Kb gene on chromosome 15q21.1, comprising nine coding exons (2-10) spanning approximately 35Kb; there are multiple first exons that are involved in tissue-specific expression [4-6]. These exons generate alternative splicing such that the coding region, and hence protein sequence, is conserved in every tissue. Within the aromatase protein, the most highly conserved region (the core region), consists of a four-helix bundle, two β sheets and the haem-binding region [7].

Aromatase deficiency is an autosomal recessive disorder due to mutations of the *CYP19A1* gene which result in reduced aromatase activity. There have been approximately twenty four cases of aromatase deficiency reported, including the original case reported by Shozu and colleagues in 1991 [8-12].

Clinical features of CYP19A1 deficiency include maternal virilization during pregnancy due to non-aromatization of foetal-derived androgens which resolves gradually postpartum, foetal virilisation of external genitalia and symptoms of haemorrhagic ovarian cysts in childhood [13-18]. Adrenarche is reportedly normal; however there is primary amenorrhoea and lack of breast development [15]. Elevated gonadotropins and hyperandrogenism are often present [16]. Virilization may progress with age – factors determining the presence and rate of progression are not established [15,19]. Delayed epiphyseal fusion and decreased bone density are reported [15,16]. Variation in phenotypic expression including some breast and uterine development may be due to partial aromatase activity. The natural history of CYP19A1 deficiency in females is largely unknown because early diagnosis leads to timely treatment. Males are usually diagnosed later in life, are tall due to delayed epiphyseal closure and have osteoporosis due to impaired bone mineralisation; these clinical features are comparable to those seen in patients with oestrogen receptor mutations; altered testis size and spermatogenesis may also be due to aromatase deficiency [15,20]. A metabolic syndrome-like picture of abdominal obesity, hepatic steatosis and insulin resistance has been well described in males; in females metabolic syndrome has been noted to be more prominent in cases where oestrogen treatment was delayed until adulthood [12,21-24].

Here, we report for the first time a female patient diagnosed with aromatase deficiency during adulthood. We found this patient to have a novel mutation in exon 8 of *CYP19A1*, which broadens the spectrum of mutations underlying aromatase deficiency.

## Case presentation

**Figure 1:**
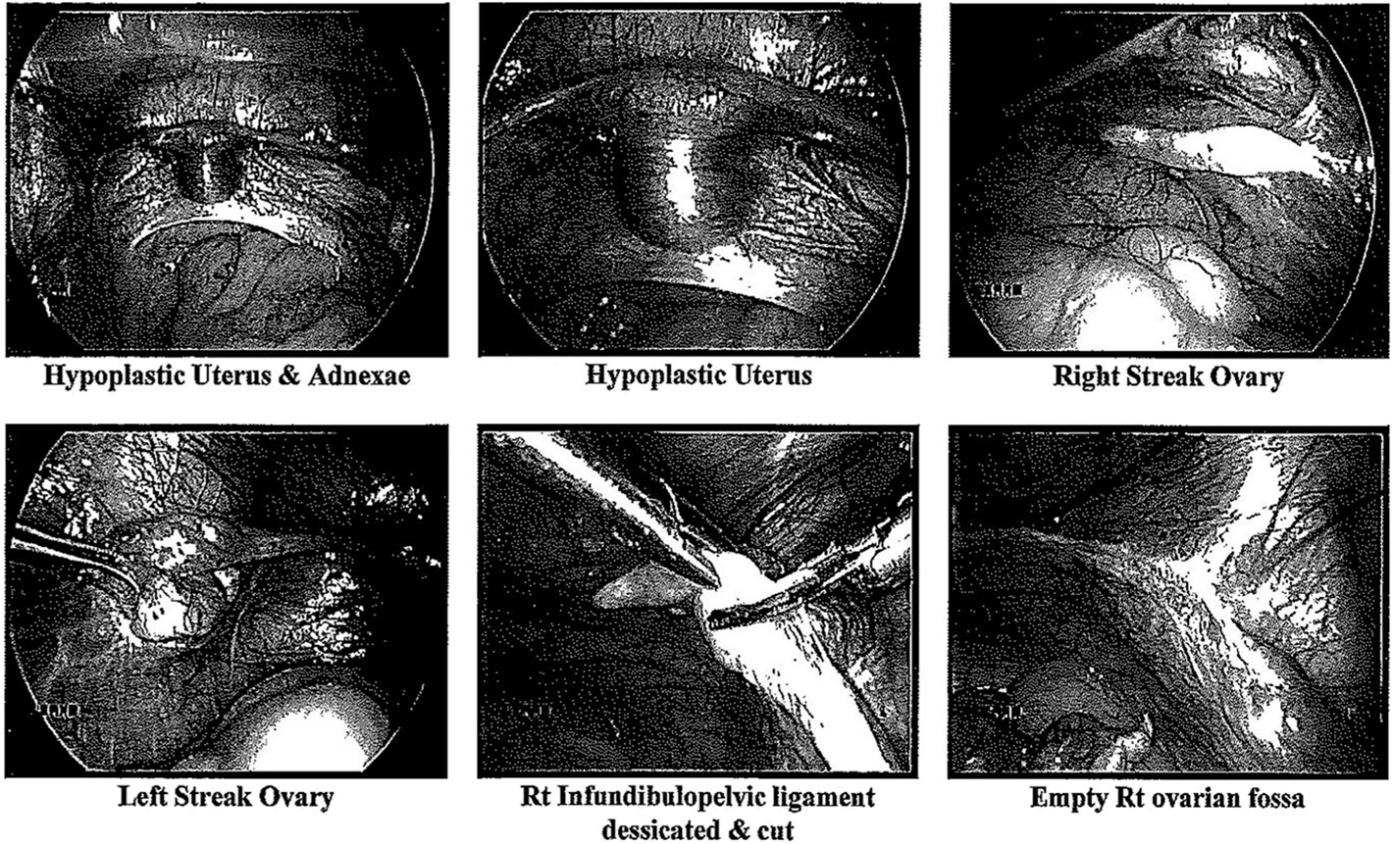
**Clinical photographs taken at the patient’s laparoscopy.** The hypoplastic uterus and adnexae, including bilateral streak ovaries are shown.

The patient was referred for endocrine review for a past diagnosis of “Turner’s syndrome”. She was born to consanguineous parents and reportedly had abnormal genitalia at birth. Growth and development were unremarkable until the teenage years when spontaneous puberty did not occur. At age 25, she came to medical attention after fracturing her right radius following a fall onto her outstretched hand. Bone densitometry revealed osteoporosis (T-score spine -2.5, total hip -1.7). Oestradiol 1 mg per day, Alendronate and Calcium carbonate with Vitamin D2 were commenced. Abnormal genitalia were again noted. She was referred to a surgical centre in India, where an examination under anaesthesia and laparoscopy revealed clitoromegaly, partial fusion of urethra and vagina (Prader classification 3), hypoplastic uterus and bilateral streak ovaries which were excised (Figure 1) [25]. Histological examination of the streak ovaries revealed atretic and primordial follicles, but no evidence of ovulation.

On referral to us after migration to Australia, the patient, aged 32, reported good health other than recurrent perineal infections which had been treated with antibiotics and drainage via abscess incision on several occasions. Surgical reconstruction of the urogenital sinus is planned. Her height was 180 cm, she had predominant abdominal obesity (waist circumference 131 cm, hip circumference 115 cm) and eunuchoidal proportions (arm span 191 cm, lower segment 103 cm). Her height was well above her mid-parental predicted height of 161 cm (father 170 cm, mother 165 cm). Her only sibling, a younger brother, has a height of 193 cm at age 30, but was unavailable for detailed examination. She did not have a webbed neck, goitre or high arched palate. Her blood pressure was in the upper normal - borderline hypertensive range at 130–145/80–100 mmHg. She was not hirsute; breasts, pubic and axillary hair were normal (Tanner stage 5). Appearance of the external genitalia has been described. Her general examination was otherwise within normal limits. The results of hormonal evaluation (on oestrogen) are shown in Table 1. Biochemical testing revealed impaired fasting glucose, dyslipidaemia and insulin resistance (Table 1). Repeat densitometry revealed osteopenia (T-score spine -2.1, T-score total hip -1.0). Moderate hepatic steatosis was evident on abdominal ultrasound.

**Table 1:** Biochemical evaluation of the patient

**Test**	**Patient**	**Reference range**
Follicle stimulating hormone	32	<12 mIU/L**
Luteinizing hormone	17	<12 mIU/L**
Testosterone	0.89	<2 nmol/L
Progesterone	<2	<6 nmol/L*
Sex-hormone binding globulin	45	25-90 nmol/L
Dehydroepiandrosterone sulphate	5.2	1-8.5 μmol/L
17-hydroxyprogesterone	2.4	0.2-11.3 nmol/L
Anti-mullerian hormone	<3 pmol/L	(below 25^th^ percentile for age)
Thyroid-stimulating hormone	2.2	0.5-4 mIU/L
Free thyroxine	15	10-25 pmol/L
Oral glucose tolerance test - fasting glucose	6.1	<5.2 mmol/L
Oral glucose tolerance test – 2 hour glucose	7.5	<7.8 mmol/L
Glycosylated haemoglobin	6.7, 7.1	<5.5%
Fasting insulin	57	<12 mU/L
Total cholesterol	5.9	<5.5 mmol/L
HDL-cholesterol	1.4	1.0-2.2 mmol/L
LDL-cholesterol	3.6	<3.7 mmol/L
Triglycerides	2	0.3-2 mmol/L
Bilirubin	8	2-24 μmol/L
Gamma-glutamyl transpeptidase (GGT)	21	<60 U/L
Alkaline phosphatase (ALP)	134	30-100 U/L
Alanine aminotransferase (ALT)	41	<55 U/L
Aspartate aminotransferase (AST)	38	<45 U/L

*Hormone measurements were made whilst the patient was on oestrogen replacement.

**Reference range quoted is for the follicular phase of the menstrual cycle.

The karyotype obtained on both peripheral blood lymphocytes and cultured fibroblasts (*n* = 134) was 46, XX. Based on the clinical history, we suspected aromatase deficiency and Sanger sequenced the nine coding exons of *CYP19A1*. We also sequenced the coding exons of P450-oxidoreductase (POR), the electron donor for cytochrome P450 enzymes (including *CYP19A1*), which has been associated with a phenotype of ambiguous genitalia and, via consequent dysfunction of *CYP19A1*, could result in maternal virilisation in pregnancy [26]. Prior written, informed consent was obtained from the patient. Primers and sequencing methods are presented in Additional file 1 (Methods).

**Figure 2:**
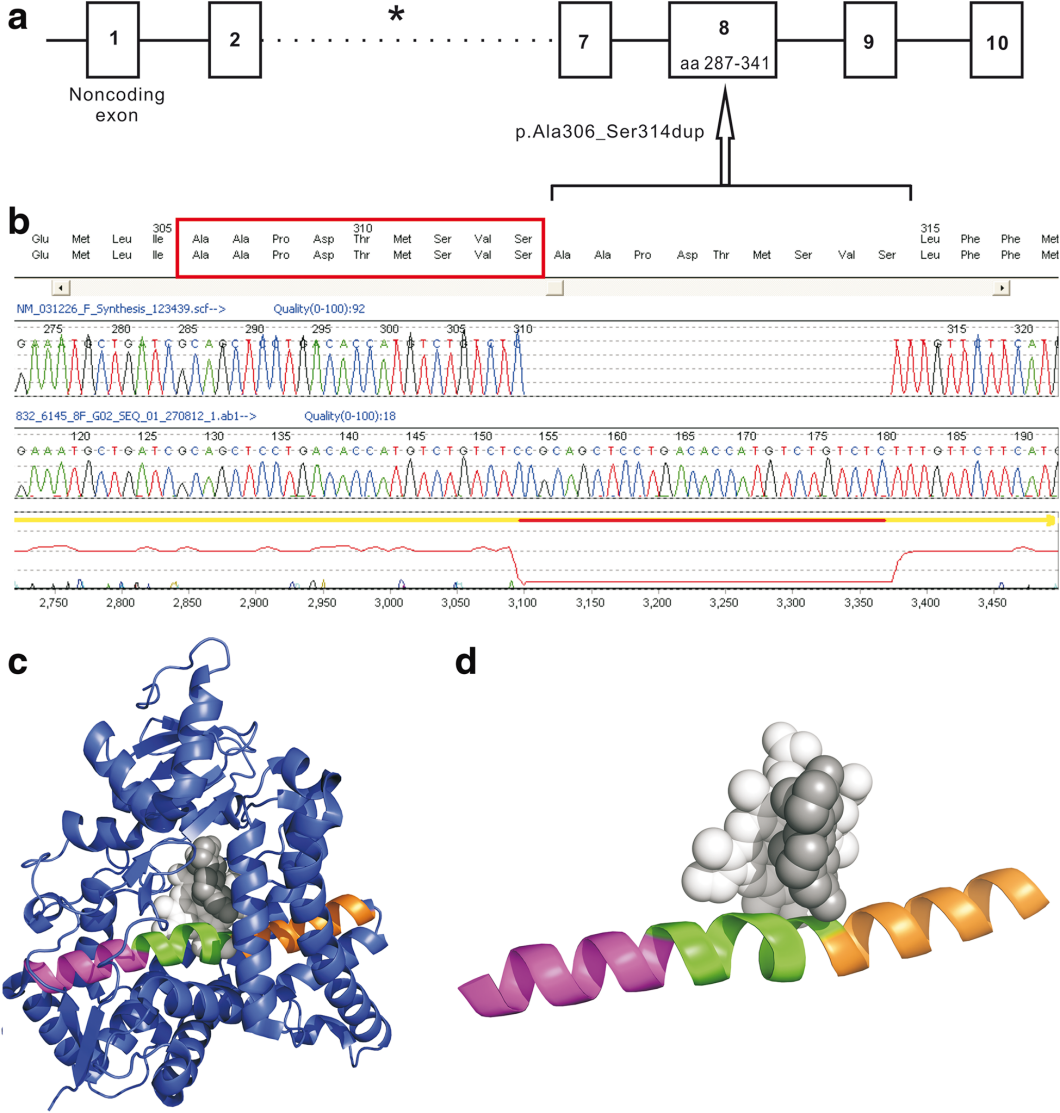
**A novel duplication of 27 base pairs in the *CYP19A1 *gene, was identified in the patient reported here.** This duplication is predicted to disrupt a critical α-helix of the aromatase enzyme. **a**. A schematic diagram of the aromatase gene, comprised of nine coding exons (exons 2-9). The location of the novel duplication identified in our patient in exon 8 is shown. **b**. Results of Sanger sequencing showing a 27 base pair duplication in exon 8 of the *CYP19A1* gene from the patient (chr15 (hg19): g.51507347_51507373dup, NM_031226.2: c.915_941dup; NP_112503.1: p.Ala306_Ser314*dup*) (visualised in Mutation Surveyor® software (Version 2.51, SoftGenetics LLC, Philadelphia, USA)). **c**. Crystallographic structure of human wild-type aromatase in complex with the cofactor protoporphyrin IX and the substrate androstenedione. The segment of an α-helix in green shows where the duplication occurs. N- and C-terminuses of the α-helix are in orange and magenta, respectively. The rest of aromatase is shown in blue. Protoporphyrin IX, white, and androstenedione, grey. The crystallographic structure is from reference 1 and visualised in PyMOL (Version 1.5.0.3, Schrödinger, LLC). A three-dimensional image of this is provided in the accompanying Additional file 2. **d**. A close-up showing the α-helix providing binding sites of protoporphyrin IX (white) and androstenedione (grey).

Sequencing of *POR* revealed two synonymous single nucleotide polymorphisms (rs1057870 and rs2228104), unrelated to the patient’s phenotype. The patient had a novel 27-base pair duplication in exon 8 of *CYP19A1* (p.Ala306_Ser314*dup*) (Figure 2a, 2b; full genomic reference presented in Additional file 1 (Results)). Whilst she is most likely to be homozygous for the observed mutation, her parents were unavailable for testing and hence we are unable to exclude hemizygosity. The *CYP19A1* mutation would be expected to result in a duplication of nine amino acids (p.Ala306_Ser314*dup*) in the protein. This duplication occurs within the aromatase α-helix, a region crucial for binding of the cofactor protoporphyrin IX and the substrate androstenedione [7,27]. The insertion of this long sequence (approximately one third of the α-helix; Figure 2c, 2d and Additional file 2) would induce a major disruption of the α-helix conformation. Prediction tools of protein secondary structure consistently suggest a break of the α-helix around the middle, turning a continuous helix into the structure of helix-coil-helix [28-30]. This would be likely to disrupt substrate and cofactor binding resulting in a lack of oestrogen synthesis (Figure 2c).

Most individuals reported with *CYP19A1* deficiency have been homozygous for loss-of-function mutations and born to consanguineous parents [8-13]. Up until recently, all reported mutations have been in coding exons, mostly in exons 9 and 10, which encode the substrate (androgen) binding site and haem-binding domains, respectively [9]. Recently, a patient with aromatase deficiency was found to be heterozygous for a novel mutation (p.Asn411Ser) in exon 9 [10]. *In vitro* studies confirmed this was a loss-of-function mutation; when co-expressed with the wildtype, the enzyme activity of the mutant was approximately 65% of wildtype; compatible with a recessive disease and thus the mutation alone did not explain the phenotype. There was a heterozygous, paternally inherited C > T variant -41 base pairs upstream of exon 1, in placental promoter I.1, a previously observed polymorphism (rs6493497) [10,31]. *In vitro* studies revealed a 50% reduction in transactivation ability of the placental promoter harbouring the mutation, compared with wildtype [10].

Our patient had several clinical features in common with the first female patient recently described with oestrogen resistance due to a novel homozygous missense mutation in a highly conserved region of exon 5 of the oestrogen receptor α (*ESR1*) [32]. Both lacked spontaneous breast development, and had primary amenorrhoea and osteopaenia. The patient with oestrogen resistance had delayed bone age and unfused epiphyses; in our patient whilst x-rays were not available we surmise from her eunuchoidal proportions that epiphyseal fusion of long bones was delayed [32]. The phenotype of tall stature, continuing linear growth into adulthood, delayed bone age, osteoporosis and eunuchoidal skeletal proportions is also well described in adult aromatase deficient males [33]. That it corrects with oestrogen treatment in both genders, reflects the common role across genders of oestrogen in bone development [34].

Our patient had a phenotype of metabolic syndrome not present in the patient with oestrogen resistance [32]. The absence of the metabolic phenotype in the latter case is in contrast with the only previously reported patient with an *ESR1* mutation [35,36]. In aromatase deficient females, the relationship between insulin resistance and oestrogen deficiency is unclear. A 14 year old female had normalisation of mild dyslipidaemia with oestrogen treatment [13]. However, a nine year old female had severe insulin resistance despite treatment with oestrogen and metformin [23]. It was postulated that *in utero* exposure to high levels of androgens or lack of oestrogens altered foetal programming of insulin sensitivity. Aromatase deficient adult men have a variable phenotype of metabolic syndrome, which improves with oestrogen treatment [14,34,37-39]. The development of central obesity post-menopausally is also considered a result of oestrogen deficiency.

The phenotype of the *CYP19A1* knockout (ArKO) mouse suggests an association between aromatase deficiency and metabolic perturbations [40,41]. Aging ArKO mice had progressively greater accumulation of visceral adiposity than wild-type, which regressed after administration of oestrogen [42]. Elevated cholesterol, insulin levels and glucose intolerance and hepatic steatosis have also been noted [42,43]. Oestrogen treatment improved glucose tolerance and reversed hepatic steatosis in male ArKO mice [43,44]. Thus, the majority of the available data infer that prolonged oestrogen deficiency is associated with a metabolic syndrome. However, there may be a gender difference in the mechanisms. High androgen levels have been associated with peripheral insulin resistance in women, but not in men [45,46]. This may be due to differences in the androgen-oestrogen ratio and its effects on cellular metabolism.

The patient we report exhibited evidence of *in utero* androgenisation of genitalia [47]. Subsequent failure of pubertal development, untreated during the adolescent years, ensued and she eventually came to medical attention with a minimal trauma fracture at age 25, prompting oestrogen replacement. She did not have features of progressive virilisation. This may reflect relatively early loss of ovarian function as a source of androgens. She had eunuchoidal proportions, a result of continued linear growth in adulthood due to unfused epiphyses in the absence of oestrogen. Genetic analyses revealed a novel 27 base duplication in exon 8 of *CYP19A1*, likely to result in a complete loss of function of the aromatase enzyme, the key enzyme regulating the oestradiol:testosterone ratios in tissues. A limitation of this work is that we have not performed functional studies to verify the effect of the duplication on protein function; however the clinical presentation and the correlation with cases already reported and with the ArKO mouse model is compelling for this being a loss of function mutation.

Unexpectedly, our patient had had streak ovaries excised. We caution that the histological slides were unavailable for verification and streak ovaries have not been described in association with aromatase deficiency. Polycystic ovaries and regression of ovarian cysts have been described previously in cases of aromatase deficiency [14-18,48]. Other cases had normal ovarian morphology, even in the absence of prior oestrogen treatment [10,11]. Thus, there is no consistent ovarian phenotype in aromatase deficiency and, furthermore, the phenotype may be modulated by treatment. In our patient, the ovarian morphology may be a result of oestrogen treatment.

Alternatively, extrapolating from observations in the ArKO mouse, we speculate that the streak ovaries may be an inherent manifestation of *CYP19A1* deficiency [40,41]. Follicular development in ArKO is abnormal in an age dependent manner, with an early block in follicular development at the antral stage with absent corpora lutea, then haemorrhagic cysts and later, absent secondary and antral follicles and atresia of primary follicles with increased collagen deposition [40,41]. The longer life span of the human may permit more complete follicular atresia and collagen deposition mimicking the classical streak ovaries seen in Turner’s syndrome. Streak ovaries may not have been previously described in *CYP19A1* deficiency because of infrequent laparoscopic visualization of the ovaries and some of the cases may have been too young to develop this manifestation. In addition, some of the cases may have had *CYP19A1* mutations that allowed some residual aromatase function with a less severe ovarian atretic phenotype.

Another possibility is that our patient may have another cause of the streak ovaries such as XO mosaicism localised to the ovaries, although the normal XX karyotype makes this less likely. Streak ovaries are not clearly associated with any other disorder but Turner’s syndrome. In view of the small numbers of CYP19A1 deficiency cases reported, this manifestation should be noted particularly since this finding may be relevant to ovarian developmental biology.

## Conclusions

We report a case of the very rare condition, aromatase deficiency, due to a novel aromatase mutation and corresponding enzyme region distinct from previous reports. Our case is unique because it is the first reported case of aromatase deficiency in a female not treated until adulthood, and it depicts, for the first time, the natural history of aromatase deficiency in females. This comprises a phenotype of osteopaenia, tall stature and metabolic syndrome, as occurs in aromatase deficient males in whom, similarly to our patient, diagnosis and treatment are delayed. Streak ovaries may represent an extension of the clinical spectrum of this disorder and appears to correlate with findings in the aromatase knockout mouse model.

### Consent

Written informed consent was obtained from the patient for publication of this Case report. A copy of the written consent is available for review by the Editor of this journal.

## Abbreviations

CYP19A1: Aromatase gene.

## Competing interests

The authors declare that they have no competing interests.

## Authors’ contributions

LG carried out the sequencing of the aromatase gene and drafted and revised the manuscript. HSS participated in the study design and drafted the manuscript. JF performed the protein prediction work, generated the figures of the protein structure presented in the manuscript and drafted the manuscript. DJT performed the clinical work. All authors read and approved the final manuscript.

## Pre-publication history

The pre-publication history for this paper can be accessed here:

http://www.biomedcentral.com/1472-6823/14/16/prepub

## Supplementary Material

Additional file 1 Methods, Results. Click here for file

Additional file 2 A three-dimensional image of the human wild-type aromatase in complex with the cofactor protoporphyrin IX and the substrate androstenedione. Click here for file
