# Supplementary material for: A case of Aromatase deficiency due to a novel CYP19A1 mutation
Source: BMC Endocr Disord. 2014 Feb 19;14:16. doi: 10.1186/1472-6823-14-16 (PMC3936939; doi:10.1186/1472-6823-14-16)
Supplement: Additional file 1 — Methods, Results. [file 1472-6823-14-16-S1.doc]

**Supplementary Methods**

**Primer design and sequence**

Primers were designed using Primer 3 (v.0.4.0) (<http://frodo.wi.mit.edu/primer3/>). The primers were selected for the following criteria: primer length 20-28, optimal 24; primer melting temperature 57ºC-63ºC, optimal 60ºC; primer GC% 30%-75%.

| **Oligo** | **Sequence - 5'-3'** | **Product size** |
| --- | --- | --- |
| CYP19ex2F | agcaacaggagctatagatgaacc | 310 |
| CYP19ex2R | atggaccaaaatcccaagtaaata | 310 |
| CYP19ex3F | aacagttgcaatttttggtgtaa | 313 |
| CYP19ex3R | agcgttagaaacaaagacatcaag | 313 |
| CYP19ex4F | agtcaaaatgtaccaccaggagtt | 348 |
| CYP19ex4R | agagtcagagcctgtctcaataaa | 348 |
| CYP19ex5F | tgtggacaattaagctccaacata | 338 |
| CYP19ex5R | cctcctagctccttgttcagtaaa | 338 |
| CYP19ex6F | cttaggctcacattttgctcaact | 318 |
| CYP19ex6R | catcagcaacttaatcaacagctc | 318 |
| CYP19ex7F | ggcaaataaatctgtttcgctaga | 271 |
| CYP19ex7R | caacagttacaaaaggggatcttt | 271 |
| CYP19ex8F | attttgttgaggttgttgatcctt | 328 |
| CYP19ex8R | ggacataagaaatggacattcaga | 328 |
| CYP19ex9F | gctttaataccaatcacagatgga | 374 |
| CYP19ex9R | ccagagaggatttaacagttgaca | 374 |
| CYP19ex10F | tgaatcaaacagagactgagtgac | 598 |
| CYP19ex10R | atttggtggaatcgggtctt | 598 |

**Polymerase chain reaction (PCR)**

Each reaction comprised:

2X LC480 ProbeMaster (Roche Diagnostics, GmbH, Mannheim, Germany) 10μL

Forward primer (10μM) 0.5μL

Reverse primer (10μM) 0.5μL

PCR-grade H_2_O 8μL

DNA (10ng/μL) 1μL

The PCR reactions were performed in the MJ Research PTC-200 thermal cycler (GMI, Inc, Minnesota, United States). The protocol was as follows:

Step 1: 95°C for 10 minutes

Step 2: 95°C for 30 seconds

Step 3: 68°C-60°C for 30 seconds – decrease by 0.8°C per cycle

Step 4: 72°C for 1 minute

Step 5: Return to Step 2, 9 times

Step 6: 95°C for 30 seconds

Step 7: 60°C for 30 seconds

Step 8: 72°C for 1 minute

Step 9: Return to Step 6, 29 times

Step 10: 72°C for 8 minutes

Step 11: 4°C forever

Step 12: End

*PCR clean-up and Sequencing reaction*

The PCR products were cleaned using ExoSAP-IT® (USB Corporation, Cleveland, Ohio, United States), according to the manufacturer’s protocol and then submitted to the Sequencing Department, Department of Genetics and Molecular Pathology, Institute of Medical and Veterinary Science, for sequencing.

*Sequencing analysis*

The sequence was analysed using Mutation Surveyor® software (Version 2.51, SoftGenetics LLC, Philadelphia, USA). Comparison was made to the reference sequence in GenBank (<http://www.ncbi.nlm.nih.gov/genbank/>).
